# Malignant Hypertension: Ocular Manifestations

**DOI:** 10.1100/tsw.2006.28

**Published:** 2006-01-26

**Authors:** Silvia Muñoz, Nieves Martín, Jorge Arruga

**Affiliations:** ^1^ Ophthalmology Department, Hospital Universitari Bellvitge, Barcelona, Spain; ^2^ Ophthalmology Department, Hospital Universitari Vall d'Hebró, Barcelona, Spain; ^3^ Ophthalmology Department, Hospital Universitari Sagrat Cor, Institut català de Retina, Barcelona, Spain

**Keywords:** Optic disk swelling, cotton-wool spots, malignant hypertension, hypertensive neuro-retinopathy

## Abstract

Malignant hypertension may be the first manifestation of systemic hypertension. We report a clinical case of a Caucasian 41-year-old man with no previous history of blood hypertension seen at casualty because of blurred vision. Fundus examination disclosed optic disk swelling, retinal hemorrhages and infarcts. The blood pressure was 220/130 mmHg. After the appropriate management of hypertension, optic disk and retinal edema resolved, leaving minor changes as mild optic disk pallor and hard exudates.

